# Prolonged Idasanutlin (RG7388) Treatment Leads to the Generation of p53-Mutated Cells

**DOI:** 10.3390/cancers10110396

**Published:** 2018-10-24

**Authors:** Lukasz Skalniak, Justyna Kocik, Justyna Polak, Anna Skalniak, Monika Rak, Agnieszka Wolnicka-Glubisz, Tad A. Holak

**Affiliations:** 1 Department of Organic Chemistry, Faculty of Chemistry, Jagiellonian University, Gronostajowa 2, 30-387 Krakow, Poland; justyna.kocik@doctoral.uj.edu.pl (J.K.); justynapolakwtf@gmail.com (J.P.); holak@chemia.uj.edu.pl (T.A.H.); 2 Department of Endocrinology, Medical Faculty, Jagiellonian University Medical College, Kopernika 17, 31-501 Krakow, Poland; anna.skalniak@uj.edu.pl; 3 Department of Cell Biology, Faculty of Biochemistry, Biophysics and Biotechnology, Jagiellonian University, Gronostajowa 7, 30-387 Krakow, Poland; monika.rak@uj.edu.pl; 4 Department of Biophysics, Faculty of Biochemistry, Biophysics and Biotechnology, Jagiellonian University, Gronostajowa 7, 30-387 Krakow, Poland; a.wolnicka-glubisz@uj.edu.pl

**Keywords:** MDM2 antagonists, idasanutlin, RG7388, p53 induction, cancer, secondary drug resistance

## Abstract

The protein p53 protects the organism against carcinogenic events by the induction of cell cycle arrest and DNA repair program upon DNA damage. Virtually all cancers inactivate p53 either by mutations/deletions of the *TP53* gene or by boosting negative regulation of p53 activity. The overexpression of MDM2 protein is one of the most common mechanisms utilized by p53^wt^ cancers to keep p53 inactive. Inhibition of MDM2 action by its antagonists has proved its anticancer potential in vitro and is now tested in clinical trials. However, the prolonged treatment of p53^wt^ cells with MDM2 antagonists leads to the development of secondary resistance, as shown first for Nutlin-3a, and later for three other small molecules. In the present study, we show that secondary resistance occurs also after treatment of p53^wt^ cells with idasanutlin (RG7388, RO5503781), which is the only MDM2 antagonist that has passed phase II and entered phase III clinical trials, so far. Idasanutlin strongly activates p53, as evidenced by the induction of p21 expression and potent cell cycle arrest in all the three cell lines tested, i.e., MCF-7, U-2 OS, and SJSA-1. Notably, apoptosis was induced only in SJSA-1 cells, while MCF-7 and U-2 OS cells were able to restore the proliferation upon the removal of idasanutlin. Moreover, idasanutlin-treated U-2 OS cells could be cultured for long time periods in the presence of the drug. This prolonged treatment led to the generation of p53-mutated resistant cell populations. This resistance was generated de novo, as evidenced by the utilization of monoclonal U-2 OS subpopulations. Thus, although idasanutlin presents much improved activities compared to its precursor, it displays the similar weaknesses, which are limited elimination of cancer cells and the generation of p53-mutated drug-resistant subpopulations.

## 1. Introduction

The tumor suppressor protein p53 is a key player engaged in the cellular response to mutations occurring within genomic DNA. Upon DNA damage, p53 induces the expression of a panel of genes responsible for the induction of cell cycle arrest and DNA repair program [1]. If the repair machinery fails, p53 induces apoptosis [2]. This allows for the controlled removal of cells which have suffered extensive, unfixable DNA damage and, thus, protects the organism against early carcinogenic events.

Considering the properties of p53 protein, it is not surprising that in almost 100% of cancers p53 functioning is impaired [3]. It is estimated that 50% of cancers deal with “inconvenient” properties of p53 by gaining a loss-of-function mutation or deletions in the *TP53* gene [4,5]. The remaining 50% express wild-type p53 protein (p53^wt^). However, the protein is restrained by its cellular negative regulators, boosted to an extent which disallows its activation. In this regard, the increase of MDM2 protein levels is the most common mechanism to inactivate p53 [3]. MDM2 has the ability to inhibit p53 functions by (i) masking its transactivation domain; (ii) targeting p53 to nuclear export; and (iii) direct ubiquitination of p53 and targeting the protein to proteasomal degradation [6].

As a consequence of the extremely frequent overexpression of MDM2 in cancer cells, the restoration of p53 functioning with MDM2 antagonists has become a promising strategy for the treatment of p53^wt^ cancers [3]. During the last years several chemical agents have been proposed to disrupt MDM2-p53 complexes, but only few compounds entered clinical trials (reviewed in [7]). Those include Nutlin-3a and its second-generation successors, RG7112 and RG7388 from Roche [8,9], AMG232 from Amgen [10,11], CGM097 and HDM201 from Novartis [12,13], SAR405838 from Sanofi [14], MK-8242 [15], DS-3032b [16], and a stapled peptide, ALRN-6924 [17]. Among these, RG7388 (idasanutlin, RO5503781) has now reached phase III clinical trials in combination with cytarabine in patients with relapsed or refractory acute myeloid leukemia [7].

The reactivation of p53 with MDM2 antagonists is a non-genotoxic strategy for the treatment of p53^wt^ tumors. Therefore, it was initially believed that this approach would be free from the development of stable secondary drug resistance. However, soon it became clear that prolonged treatment with Nutlin-3a, which was the first active MDM2 antagonist, results in the appearance of p53-mutated drug-resistant clones [18,19,20]. In the recent years, this phenomenon was reported also for some further potent MDM2 antagonists: SAR405838 [21,22], MI-63 [23], and HDM201 [24]. However, so far no such data has been provided for the most advanced antagonist, idasanutlin. Therefore, in this report, we explore the issue of limited elimination of p53^wt^ cancer cells by idasanutlin and provide proofs for the de novo development of *TP53*-mutated drug-resistant cell populations out of idasanutlin-responsive p53^wt^ U-2 OS cells.

## 2. Results

### 2.1. Idasanutlin Activates p53 and Induces Cell Cycle Arrest in Four Model p53^wt^ Cancer Cell Lines

To evaluate the activity of idasanutlin, three human p53^wt^ cancer cell lines were chosen, which are commonly used for studying the activity of MDM2 antagonists. These were two osteosarcoma cell lines—U-2 OS and SJSA-1, and MCF-7, a human breast adenocarcinoma cell line. Additionally, p53^del^ human osteosarcoma cell line SAOS-2 was used as a control of treatment specificity.

**Figure 1:**
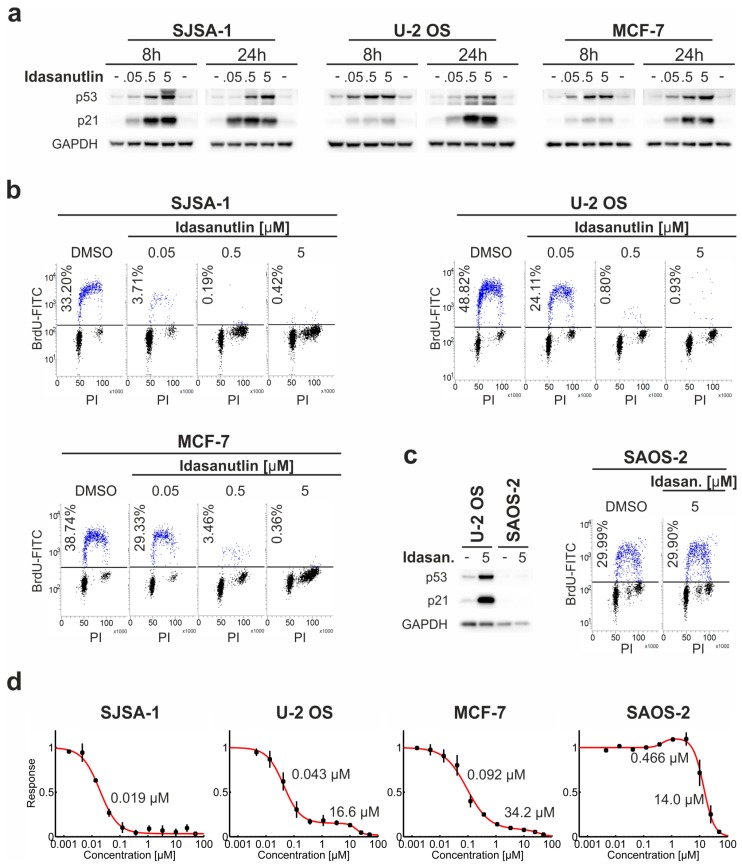
Idasanutlin activates p53 and inhibits the growth of p53^wt^ cells. (**a**–**c**) Three p53^wt^ cell lines, SJSA-1, U-2 OS, and MCF-7, and one p53^del^ cell line, SAOS-2, were treated for 8 or 24 h with 0.05, 0.5, or 5 μM idasanutlin, or DMSO as a control. Western blot detection of p53, p21, and GAPDH was performed for the cells treated for 8 and 24 h. Cell cycle analysis was performed following 24 h of treatment with BrdU pulse-labeling for the last hour. The cells were fixed and stained with propidium iodide (PI) and FITC-anti-BrdU antibody (BrdU-FITC). The results presented on panels (**a**–**c**) are representative of at least three independent experiments. (**d**) SJSA-1, U-2 OS, MCF-7, and SAOS-2 cells were treated for five days with increasing concentrations of idasanutlin or an equivalent volume of DMSO, before assessment of cell viability. The data were analyzed with Dr-Fit software [26] and the best fitting model (red line) was chosen based on the Bayesian Information Criterion (BIC) (Supplementary Table S1). EC_50_ values of the growth inhibitory/stimulatory effects determined by the Dr-Fit software are indicated (see also Supplementary Table S1). Data points show means ± SD from three independent experiments.

Idasanutlin strongly increased p53 expression already at 0.05 µM concentration in all three p53^wt^ cell lines (Figure 1a). Additionally, a potent increase of the expression of cyclin-dependent kinase inhibitor 1 (p21) was observed, providing evidence of functional activation of p53 (Figure 1a, [25]). As a result of p53 activation, a strong cell cycle arrest of the treated cells was observed already at the lowest concentration of idasanutlin (0.05 µM) and reached nearly 100% of the arrested cells at higher concentrations (Figure 1b). Idasanutlin was inactive in SAOS-2 cells, which do not express p53 protein (Figure 1c).

Idasanutlin also increased the expression of MDM2 protein in the two *MDM2*-amplified osteosarcoma cell lines used in this study, i.e., SJSA-1 and U-2 OS (Supplementary Figure S1). For U-2 OS cells a successive, but low induction of MDM2 protein expression was observed in response to idasanutlin, while for SJSA-1 cells the induction was very strong and rapid, but decreased with time, reaching the level of control at 48 h of the treatment (Supplementary Figure S1).

### 2.2. Dose-Dependent Response to Idasanutlin Is Biphasic, Revealing Two Inhibitory Effects on Cell Survival

To have a deeper view into the growth inhibitory effect of idasanutlin, cells were seeded at low confluence and treated for five days with increasing concentrations of the drug. In accordance with the cell cycle analysis results, the growth of all four cell lines was largely affected by idasanutlin, as measured by MTT cell survival assay (Figure 1d). In order to choose the best model describing the experimental data, a dose-response fitting software, Dr-Fit, developed recently by Di Veroli and co-workers, was used [26].

For SJSA-1 cells, a single, strong drop-down of cell viability was observed with EC_50_ value of 0.019 µM, confirming the elimination of these cancer cells by idasanutlin (Figure 1d, Supplementary Table S1). For the remaining cells, dose-response curves had clear non-monophasic courses. Indeed, for U-2 OS and MCF-7 cells, a biphasic, two inhibitory dose-response relationship was suggested by the software (Figure 1d, Supplementary Table S1). A low concentration effect was observed with EC_50_ values in the range of 0.043–0.092 µM, and a high concentration effect with EC_50_ values of 16.6 µM for U-2 OS cells and 34.2 µM for MCF-7 cells. The high concentration effect likely reflects unspecific toxicity of the compound, since at concentrations above 10 µM, also the survival of p53^del^ SAOS-2 cells was largely affected (Figure 1d). In contrast, at low concentrations, the effect was observed only for p53^wt^ cells and thus can be attributed to the specific activity of p53 protein. Importantly, in the range of p53-specific activity of idasanutlin (0.01–10 µM), U-2 OS and MCF-7 cells were not eliminated but only stalled in the cell cycle. Even after five days, U-2 OS cell populations treated with idasanutlin at concentrations up to 10 µM were composed of metabolically active cells that contained integral, round-shaped nuclei devoid of any symptoms of necrotic or apoptotic death (Supplementary Figure S2).

For p53^del^ SAOS-2 cells, an initial stimulatory effect just preceding the inhibitory drug-toxic effect was detected (Figure 1d). This may be explained by some hermetic effects [27] evoked by idasanutlin in p53^del^ cancer cells.

### 2.3. Idasanutlin Fails to Induce Apoptosis in U-2 OS and MCF-7 Cells

**Figure 2:**
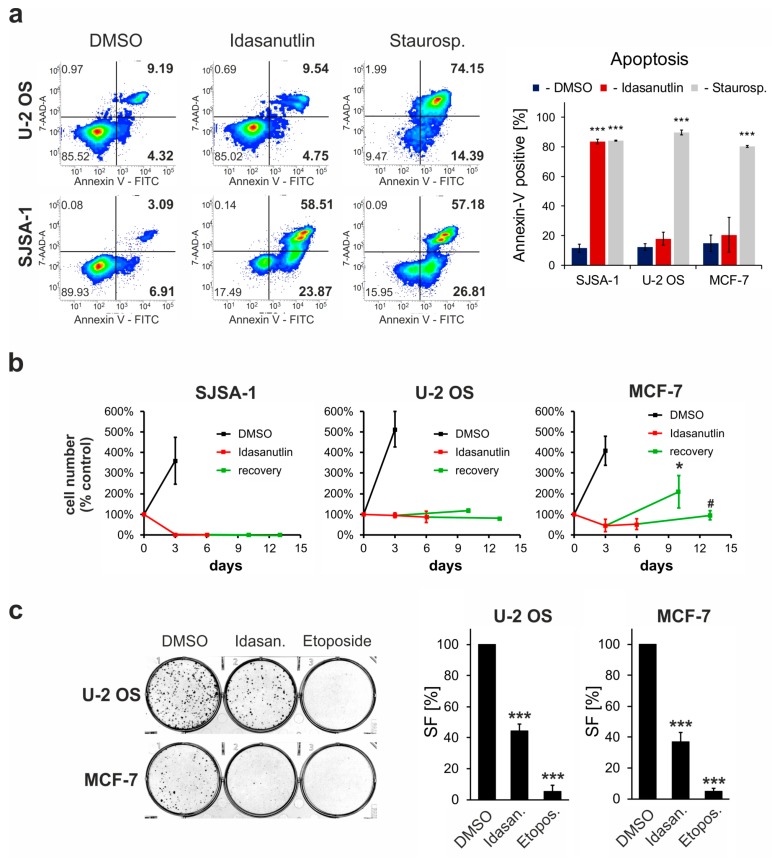
The insufficient elimination of p53^wt^ cancer cells by idasanutlin. (**a**) Apoptosis detection. U-2 OS, SJSA-1, and MCF-7 cells were treated with DMSO or idasanutlin for 48 h, or with staurosporine for 16 h. The detection of apoptosis was performed by flow cytometry analysis following cell staining with 7-AAD and FITC-conjugated annexin V. The plots present representative results for U-2 OS and SJSA-1 cells, and the graph shows mean ± SD values from three independent experiments. The statistical significance was evaluated using a *t*-test: *** *p* < 0.001. (**b**) Treatment/recovery assay. SJSA-1, U-2 OS, and MCF-7 cells were treated with DMSO for 3 days, or with 5 µM idasanutlin for three or six days, followed by washing and a seven-day recovery period in fresh cell culture medium. The cells were fixed at time points three days, 3 + 7 days, six days and 6 + 7 days, stained with Hoechst 33342, and pictured for the calculation of cell nuclei. Each data point comprises the mean ± SD value from three independent experiments (*n* = 3) with three images quantified in every experiment. The statistical significance was evaluated using a *t*-test: * *p* < 0.05 and # *p* < 0.05 seven days recovery after three or six days of the treatment versus three or six days treatment, respectively. (**c**) Colony formation assay. U-2 OS and MCF-7 cells were treated with DMSO, 5 µM idasanutlin, or 1 µM etoposide for four days, seeded (500 cells per well) on six-well plates and cultured for an additional seven days without drugs. The colonies were visualized by crystal violet staining and surviving fraction (SF) values versus DMSO-treated controls were calculated. The graph shows mean ± SEM values from three independent experiments. The statistical significance was evaluated using ANOVA with Tukey’s post-hoc test: *** *p* < 0.001 vs. DMSO-treated control.

Activated p53 is able to induce apoptosis under specific circumstances [2]. In several studies, MDM2 antagonists have been shown to induce diverse pro-apoptotic effects in different p53^wt^ cell lines. To test the induction of apoptosis in the chosen cells, FITC-Annexin V/7-AAD double staining was performed. As a positive control, Staurosporine (1 µM) was used, leading to strong apoptosis in all three cell lines (Figure 2a). In contrast, idasanutlin induced apoptosis only in SJSA-1 cells, which are known to be highly sensitive to MDM2 antagonists, but not in U-2 OS or MCF-7 (Figure 2a). Thus, although cell cycle arrest is a common reaction to idasanutlin leading to growth inhibition, apoptosis is limited to a subset of p53^wt^ cancer cells.

### 2.4. p53^wt^ cells Remain Viable Following the Idasanutlin Treatment

A biphasic, two inhibitory course of cell survival curves together with cell cycle arrest and lack of apoptosis in U-2 OS and MCF-7 cells suggest that the cells may remain viable even after prolonged treatment with idasanutlin. To verify this hypothesis, two experimental approaches were undertaken: treatment-recovery assay and colony-formation assay.

For the treatment-recovery assay, the cells were first treated with 5 µM idasanutlin for three or six days, gently washed with fresh medium and cultured in the absence of the drug for an additional seven days. To adequately evaluate the growth of the cell population, cell numbers were calculated following nuclei staining with Hoechst 33342. In accordance with cell cycle, apoptosis, and MTT assays, idasanutlin eliminated almost all SJSA-1 cells and potently blocked the growth of U-2 OS and MCF-7 cells (Figure 2b). Most importantly, for the U-2 OS cell line, the number of the cells was constant throughout the experiment, and for MCF-7 cells after the initial drop-down of the populations, which may be due to non-apoptotic cell death, its reproduction over the initial cell number was observed during the recovery period (Figure 2b). The viability of idasanutlin-treated U-2 OS and MCF-7 cells was confirmed by the colony-formation assay. Although a relatively harsh idasanutlin treatment was applied (5 µM idasanutlin, 4 days of the treatment), around 40% of the cells were still able to form colonies when transferred to fresh cell culture plates and media (Figure 2c). This demonstrates that the growth-inhibitory action of the drug is reversible. In contrary, similar treatment with 1 µM etoposide almost completely blocked colony-forming potential of the treated cells (Figure 2c).

### 2.5. Continuous Treatment of U-2 OS Cells with Idasanutlin Leads to the Selection of p53-Mutated Drug-Resistant Clones

**Figure 3:**
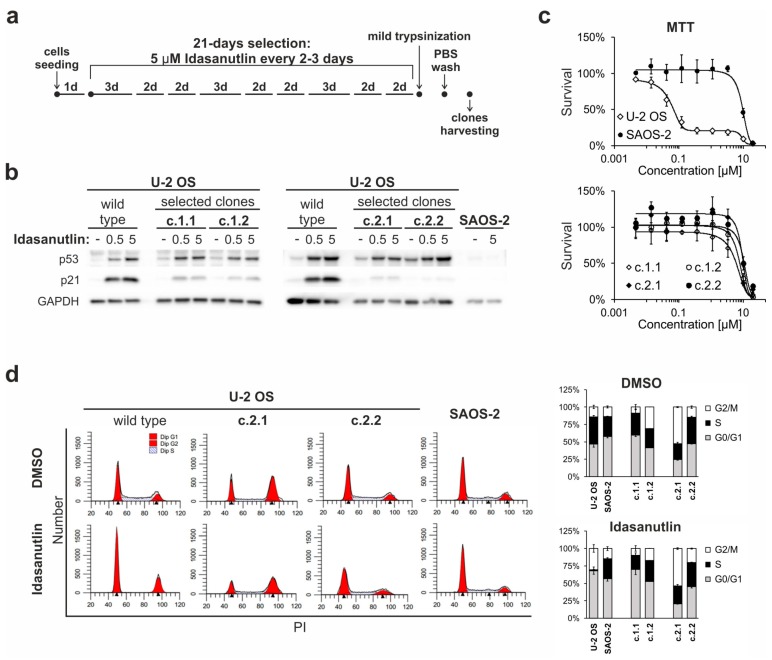
The selection of idasanutlin-resistant subpopulations of U-2 OS cells. (**a**) Selection strategy scheme. U-2 OS cells were seeded on 75 cm^2^ cell culture flasks at sub-confluency. The next day, treatment with 5 µM idasanutlin was performed and repeated every 2–3 days, as indicated. After 21 days of the treatment, idasanutlin-sensitive cells (not-proliferating) were removed by mild trypsinization, and clones were picked and cultured individually. (**b**) Induction of p53 and p21 expression in idasanutlin-resistant clones. U-2 OS and SAOS-2 cells, as well as four idasanutlin-resistant clones (c.1.1, c.1.2, c.2.1, and c.2.2) were treated for 24 h with 0.5 or 5 µM idasanutlin, or DMSO as a control, followed by Western blot detection of p53, p21, and GAPDH expression. The presented results are representative of 3 independent experiments. (**c**) MTT cell survival test was performed on U-2 OS and SAOS-2 cells, and idasanutlin-resistant clones. The cells were treated for five days with increasing concentrations of idasanutlin or an equivalent volume of DMSO. The data represent mean ± SD values from three independent experiments, each performed in duplicates, and are presented as % of DMSO-treated control. (**d**) Cell cycle analysis of DMSO- or idasanutlin-treated U-2 OS cells, SAOS-2 cells and idasanutlin-resistant clones (c.2.1 and c.2.2) was performed after 24-h treatment with 5 µM idasanutlin or DMSO by propidium iodide (PI) staining. Graphs present cell cycle distribution as mean ± SD values from three independent experiments.

Based on the results described above, U-2 OS cells were chosen to test the outcome of a long-term treatment with idasanutlin. The aim of this experiment was to check whether the prolonged exposure to the drug leads to the elimination of p53^wt^ cells, or to the generation of secondary drug resistance. For this purpose, the cells were seeded at sub-confluency and cultured in the constant presence of 5 µM idasanutlin for 21 days. Fresh portions of idasanutlin were applied every 2–3 days, each time the culture medium was exchanged (Figure 3a).

While the morphology of the treated cells changed throughout the experiment, the treatment did not result in a massive death of U-2 OS cells. Meanwhile, the formation of colonies of densely-packed proliferating, presumably idasanutlin-resistant cells was observed. The clones were picked, re-populated and subjected to further analyses. The clones were also subjected to authentication by multiplex analysis of short tandem repeats (STR) at 15 loci. The authentication confirmed that the clones originated from the U-2 OS cell line.

Four clones selected in two separate experiment repeats were chosen for deeper characterization. Western blot analysis revealed that all clones expressed the p53 protein. The treatment with idasanutlin increased the expression of p53 to an extent comparable to the parental U-2 OS cells (Figure 3b). However, the induction of p21 expression in the clones was significantly impaired, suggesting defective functioning of the p53 protein (Figure 3b). Accordingly, the clones occurred to be resistant to idasanutlin treatment, as shown by cell survival MTT assay, where they presented a response similar to the p53^del^ SAOS-2 cells (Figure 3c). This was further supported by flow cytometry analysis of cell cycle distribution. While the treatment with idasanutlin resulted in an almost complete arrest of U-2 OS cells, the U-2 OS-derived clones retained proliferative potential in the presence of the drug (Figure 3d). Altogether, the results suggested that the selected clones were resistant to idasanutlin.

Cells with acquired resistance to other MDM2 antagonists have been shown to bear mutations in the p53-encoding *TP53* gene [18,19,22,23]. To check whether the resistance to idasanutlin is also related to DNA mutations, exons 5–8 of the *TP53* gene (LRG_321 t1) were sequenced and analyzed for any genetic alterations. For each of the four sequenced clones, the presence of a mutation in the *TP53* gene was detected, each in heterozygous configuration with the wild-type allele. For the clones c.1.1, c.2.1, and c.2.2 the revealed point mutations, rs786202962 (c.527G>A), rs587781991 (c.404G>A), and rs876660807 (c.715A>G), respectively, are of conflicting pathogenic significance or likely pathogenic, according to annotations at the NCBI ClinVar database (ID 186451, ID 141762, and ID 234036, respectively) [28]. According to the UMD mutation database, those mutations (rs786202962 (UMD_3138), rs587781991 (UMD_2048), and rs876660807 (UMD_4640)), are located in highly-conserved domains, responsible for DNA binding, and are predicted to be damaging [29]. For the clone c.1.2, a six-nucleotide deletion (c.575_580del) was detected in a region frequently mutated in cancers, presumably resulting in the expression of p53 protein with deleted amino acids 192 and 193. This mutation was assigned by *SIFT INDEL* prediction as damaging to p53 protein function [30]. In the UMD database, this rare variant (UMD_3519) is summarized as likely pathogenic, although the consequences of this mutation on p53 protein activity are unknown. This result gives the rationale for the observed resistance to idasanutlin.

### 2.6. Idasanutlin Resistance Is Newly Developed from Initially Sensitive, Monoclonal Cell Populations

Drug resistance may be developed de novo during treatment by the acquisition of genomic or epigenomic alterations or may be the result of the selection of subpopulations of resistant cells pre-existing in the initial cell populations. To determine the ability of U-2 OS cells to acquire resistance to idasanutlin during treatment, monoclonal populations of U-2 OS cells (derived from a single cell) were developed by seeding the cells at very low concentration (in average 0.5 cell per well) and re-populating the resulting colonies. This strategy ensured a homogenous genetic background of all cells within every separate clonal population. These clonal populations were then subjected to idasanutlin selection for 12 days, followed by BrdU labelling and staining with FITC-conjugated anti-BrdU antibody and Hoechst in a search for proliferating, drug-resistant colonies.

**Figure 4:**
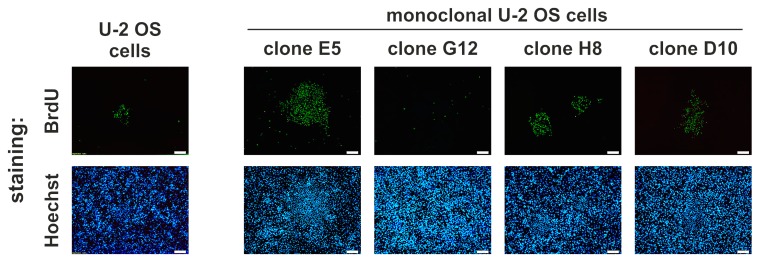
The development of idasanutlin-resistant cell populations from monoclonal U-2 OS cells. Monoclonal U-2 OS cell populations named E5, G12, H8, and D10 (each derived from a single U-2 OS cell), and parental U-2 OS cells were treated with 5 µM idasanutlin for 12 days, with new portions of the drug added every 2–3 days, whenever the medium was exchanged. During the last 24 h, the cells were labeled with BrdU. Then, the cells were fixed and double-stained with anti-BrdU FITC-conjugated antibody (proliferating cells) and Hoechst 33342 (all cells) and visualized with fluorescence microscopy. The presented pictures are representative for the clones, found for each cell type in two separate experiments. White scalebar: 200 µm.

The formation of idasanutlin-resistant clones was noted in 6 out of 15 tested monoclonal U-2 OS populations. Some of the examples of observed BrdU/FITC-anti-BrdU stained clones are shown in Figure 4. This undeniably shows that secondary resistance to idasanutlin is being developed during and as a result of the treatment.

**Figure 5:**
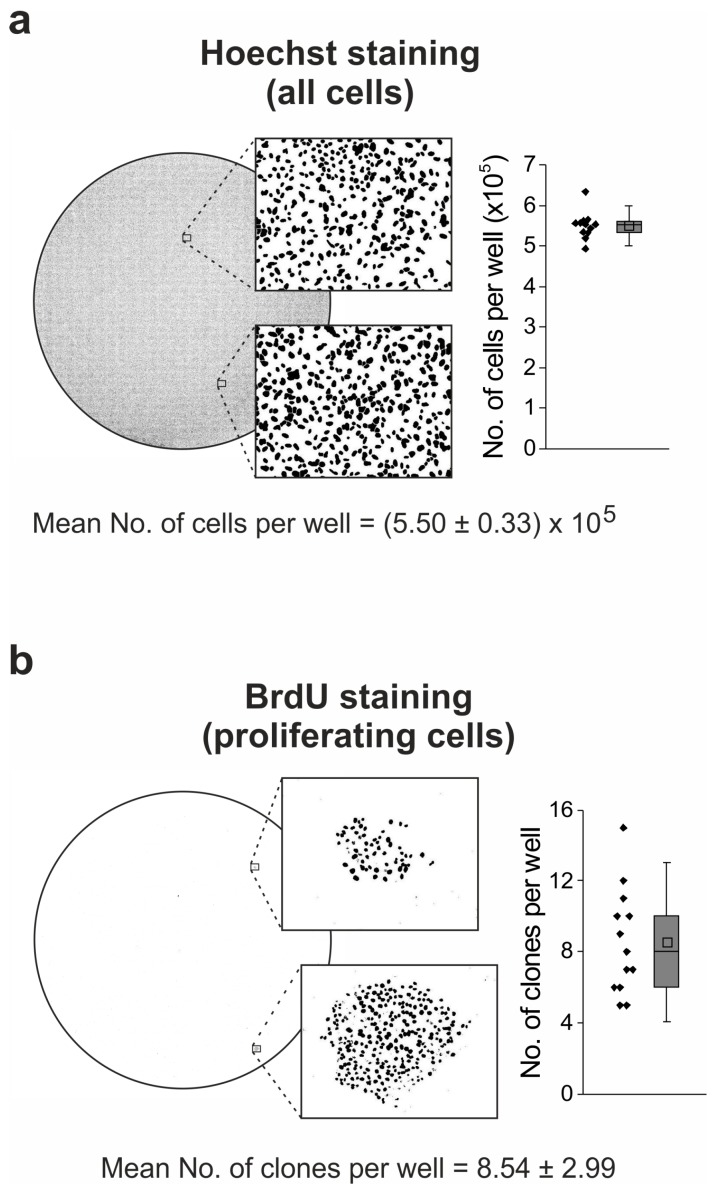
Emergence of idasanutlin-resistant clones. U-2 OS cells (5 × 10^5^) were seeded on six-well plates and treated the next day with 5 µM idasanutlin. The treatment was performed for 12 days with fresh portions of idasanutlin added every 2–3 days, whenever the medium was exchanged. During the last 24 h, the cells were labeled with BrdU. Then, the cells were fixed and stained with anti-BrdU FITC-conjugated antibody (proliferating cells) and Hoechst 33342 (all cells). Whole surfaces of every well were visualized with fluorescence microscopy using a scanning stage. Total cell numbers and total colonies numbers were calculated using ImageJ software (Hoechst-stained nuclei) or manually (FITC-stained nuclei). The experiment was performed five times with a total number of 13 analyzed wells. The presented pictures of cell nuclei are representative and were processed from initial fluorescence readouts using ImageJ in order to enable automated nuclei quantification.

Finally, we wanted to evaluate the frequency of generation of drug-resistant clones from the initial populations of U-2 OS cells. For this purpose, 5 × 10^5^ cells were seeded at six-well plates and cultured for 12 days in the presence of 5 µM idasanutlin. The medium was exchanged every 2–3 days and supplemented with fresh portions of idasanutlin. At the end of the treatment, the cells were labeled with BrdU and stained with a FITC-conjugated anti-BrdU antibody and Hoechst. The whole area of each well was then visualized with a fluorescence microscope equipped with a scanning stage (Figure 5a,b). Drug-resistant colonies of proliferating cells (Figure 5b), as well as all cells stained with Hoechst (Figure 5a), were calculated with ImageJ.

Idasanutlin treatment for 12 days did not alter the number of seeded U-2 OS cells (5 × 10^5^ seeded cells vs. mean (5.50 ± 0.33) × 10^5^ of the final cell nuclei numbers, Figure 5a). This ultimately confirmed a limited elimination of U-2 OS cells by idasanutlin. The mean amount of resistant clones, which developed during the experiment, was 8.54 ± 2.99 (Figure 5b), giving the in vitro frequency of 15.5 acquired resistance events per one million of the seeded cells.

## 3. Discussion

Up-to-date reports leave no doubts that MDM2 antagonists fulfil their intended task, leading to the disruption of MDM2-p53 complexes both in vitro and in vivo. Reactivation of p53 in p53^wt^ cancer cells is spectacular and leads to a powerful cell cycle arrest upon the treatment with MDM2 antagonists. Additionally, the limited off-target action of second generation inhibitors, such as idasanutlin or AMG232, was confirmed by comparing their cytostatic effect in cells expressing wild-type p53 versus cells deprived of p53 expression [9,11].

However, several reports indicated a lack of induction of apoptosis in multiple p53^wt^ cells treated with MDM2 antagonists. Already in 2006, Tovar and co-workers presented that although Nutlin-3a induces strong cell cycle arrest in all 10 p53^wt^ tested cell lines, in most of them the induction of apoptosis was barely observed [31]. In 2008, Paris and co-workers supported this observation [5] and one year later, Huang and colleagues showed that Nutlin-3a induces cell cycle arrest and a senescence-like state in several tested cell lines, but many of those cell lines continue to proliferate upon drug removal [32]. Finally, in 2015, the activity of CGM097, another MDM2 antagonist which is now in clinical trials, was tested on a panel of 113 p53^wt^ and 243 p53^mut^ cell lines [33]. Not surprisingly, p53^mut^ cancer cell lines were almost exclusively resistant to MDM2 antagonists. However, among p53^wt^ cells, 70 out of 113 were found insensitive to the drug [33]. Altogether, these results suggested that the presence of wild-type p53 is not sufficient for the cell lines to provide susceptibility to MDM2 antagonists and that some other factors or aberrances in the p53 pathway modulate the proapoptotic functions of the released p53.

In the different p53^wt^ cells, additional p53 regulators may block the activation of p53 and, therefore, limit the response to MDM2 antagonists. Some examples of such regulators are MDM4 [34], exportin (XPO1) [35] or nucleophosmin (NPM1) [36]. Similarly, low MDM2 levels may also cause problems due to the lack of molecular targets for the treatment, and *MDM2* gene amplification was postulated as a prognostic factor for successful MDM2 antagonist therapy. However, in the cell lines that we have picked for our study, the activation of p53 seems to be rather strong, as evidenced by western blot results presenting the induction of p21 expression levels. In fact, all cells stopped cycling when treated with idasanutlin, and the only difference was the level of apoptosis that was induced in response to the drug. This suggests that some downstream factors, rather than upstream regulators of p53, define the response to idasanutlin in these cells.

For MCF-7 cells, low apoptosis induction may be caused by the lack of caspase 3 activity in these cells [37,38]. The activation of caspase 3 has been shown to be crucial for the induction of apoptosis in p53^wt^ cells following nutlin-3 treatment [39]. U-2 OS and SJSA-1 cells both carry MDM2 gene amplifications [23,40] and there is a 53 dependent increase in MDM2 protein levels in cells treated with MDM2 antagonists, providing a negative feedback loop for p53 activity [41]. Although in both SJSA-1 and U-2 OS cells, the expression of MDM2 increased following idasanutlin treatment, our results show that that this increase differs between those two cell lines both in the extent of MDM2 expression level and the time profile of this induction. Specifically, for SJSA-1, a strong and rapid but transient induction of MDM2 expression was observed. Since the inhibition of MDM2 increases the efficiency of nutlin-induced apoptosis [42], this down-regulation of MDM2 expression in SJSA-1 cells may contribute to, or at least reflect the increased susceptibility of these cells to apoptosis, observed following idasanutlin treatment. However, further functional studies are required to verify this hypothesis.

As a consequence of insufficient apoptosis induction by MDM2 antagonists, cancer cells are given the time to gain additional mutations and eventually develop drug resistance, as reported initially for the first generation MDM2 antagonist, Nutlin-3a [18,19,20], and later for additional compounds, i.e., SAR405838 [21,22], MI-63 [23], and HDM201 [24]. In this report, we provide complementary results for idasanutlin, which is the most advanced MDM2 antagonist with respect to clinical trials, and possibly also activity. Our data clearly show that the selection of resistant cells is due to the de novo appearance of DNA mutations during the treatment and not just the selection of pre-existing cells, since resistant clones appear also in monoclonal p53^wt^ U-2 OS cell populations following treatment with idasanutlin. The frequency of drug resistance was estimated to be around 15.5 per 10^6^ cells, which is comparable to the previously published frequencies of acquired drug resistance [43]. Although in our study all four selected idasanutlin-resistance clones were found to bear deleterious mutations in the *TP53* gene, it is worth mentioning that additional mechanisms of acquired resistance to MDM2 antagonists have been reported previously. In the study by Michaelis and co-workers, nutlin-3 was found to select for both p53-mutated and p53^wt^ populations of resistant UKF-NB-3 cells, indicating that the mutation of *TP53* gene is not an exclusive way of gaining resistance to the drug [18]. In 2017, Chapeau and colleagues studied the acquisition of resistance to MDM2 antagonists HDM201 in an in vivo mouse model [24]. While 53.7% of resulting tumors had mutations in the *TP53* gene, four other genes were found to be frequently mutated, causing the resistance to HDM201. Those were *MDM4*, *BCL-xL*, *ΔNTRP63*, and *ΔNTRP73*—four genes known to regulate p53, directly or indirectly [24]. Altogether, the mentioned reports show that in order to fully benefit from the antiproliferative potential of MDM2 antagonists the issue of the generation of secondary resistance needs to be addressed.

One of the ways to deal with the generation of secondary resistance is to enhance the elimination of cancer cells by combining MDM2 antagonists with additional anticancer treatments. Indeed, such studies are being conducted since many years for Nutlin-3a, and recently also for other compounds, including idasanutlin. Synergistic effects have been reported for combining idasanutlin with X-radiation [44], chemotherapy (cisplatin, doxorubicin, topotecan, temozolomide and busulfan) [45], venetoclax, an inhibitor of Bcl-2 [46,47], GSK2830371, a WIP1 inhibitor [48,49], obinutuzumab, an anti-CD20 antibody [50], rucaparib, a PARP inhibitor [51], palbociclib, a CDK4 inhibitor [52], and BEZ235, a PI3K/mTOR dual inhibitor [53]. Additionally, some reports show that although p53^wt^ cells acquire resistance to MDM2 antagonists both in vitro and in vivo, they retain susceptibility to some other chemical and physical anticancer treatments, such as Bcl-2 inhibition [21] or X-rays [23]. This gives hope for the development of combined strategies that would not only prevent the development of secondary resistance, but also deal with cancer cells, which acquire resistance to MDM2 antagonists.

## 4. Materials and Methods

### 4.1. Cell Lines

Human osteosarcoma cell lines U-2 OS and SAOS-2, and human colorectal carcinoma cell line HCT 116 were cultured in Mc Coy’s 5A medium supplemented with 10% fetal bovine serum (FBS) (BioWest, Nuaillé, France). Human osteosarcoma cell line SJSA-1 was cultured in RPMI-1640 medium supplemented with 10% FBS. Human breast adenocarcinoma cell line MCF-7 was cultured in DMEM medium supplemented with 10% FBS. The cells were purchased from Sigma Aldrich (Saint Louis, MO, USA, original source: European Collection of Authenticated Cell Cultures) and were routinely tested for mycoplasma infection using a PCR-based method [54].

### 4.2. Western Blotting

The Western blotting procedure was performed as described before [55]. The following antibodies and dilutions were used: rabbit polyclonal anti-p53 (1:200, Santa Cruz Biotechnology, cat. sc-6243, Dallas, TX, USA), rabbit monoclonal anti-p21 (1:1000, Cell Signaling Technology, CST, cat. 2947, Danvers, MA, USA), rabbit monoclonal anti-MDM2 (1:1000, Thermo Fisher Scientific, cat. 700555, Waltham, MA, USA), rabbit monoclonal anti-GAPDH (1:4000, CST, cat. 2118, Danvers, MA, USA) and goat peroxidase-conjugated anti-rabbit (1:2000, CST, cat. 7074, Danvers, MA, USA).

### 4.3. Cell Cycle Analysis

The cells were treated with DMSO or idasanutlin (RG7388, Selleck Chemicals, Houston, TX, USA) for 24 h with pulse-labeling using 10 µM bromodeoxyuridine (BrdU, Sigma Aldrich) for the last hour of the treatment. After that, the cells were harvested by trypsinization, fixed with 96% ethanol and stored at −20 °C. The cells were centrifuged and suspended in 2 M HCl/0.5% Triton X-100, added dropwise. Following 30 min incubation at room temperature and centrifugation, the cells were resuspended in 0.1 M Na_2_B_4_O_7_, pH 8.5, centrifuged again and suspended in 0.5% Tween 20/1% BSA/PBS. FITC-conjugated anti-BrdU antibody (BioLegend, cat. 364104, San Diego, CA, USA) was then added (5 µL per 10^6^ cells) and the cells were incubated at 4 °C overnight with gentle shaking (200 rpm). The cells were centrifuged, suspended in PBS containing 5 µg/mL of propidium iodide (PI, SERVA Electrophoresis GmbH, Heidelberg, Germany) and 10 µg/mL RNase A (Thermo Scientific, Waltham, MA, USA), incubated for 20 min at room temperature, and analyzed with BD FACSVerse flow cytometer (Becton Dickinson, Franklin Lakes, NJ, USA). Cell cycle distribution was analyzed using ModFit LT Software (Verity Software House, Topsham, ME, USA).

### 4.4. Annexin V/7-AAD Staining of Apoptotic Cells

The cells were treated with DMSO or idasanutlin for 48 h, or with Staurosporine (Santa Cruz Biotechnology, Dallas, TX, USA), a well-known inducer of apoptosis, for 16 h. Following this treatment, the cells were trypsinized and collected together with growth media and PBS, which was used for washes, to avoid any loss of detached apoptotic cells. The cells were centrifuged, washed with PBS, centrifuged again, suspended with annexin-binding buffer (BioLegend) and stained with 5 µg/mL of FITC-conjugated annexin V (BioLegend) and 2.5 µg/mL 7-Aminoactinomycin D (7-AAD, BioLegend) for 15 min at room temperature. The samples were analyzed using BD FACSVerse flow cytometer (Becton Dickinson) with appropriate color compensation.

### 4.5. Cell Viability MTT Assay and Dose-Response Curve Fitting

For the MTT assay, the cells were seeded at low confluency on 96-well transparent plates (Falcon, Corning, NY, USA) and treated the next day with idasanutlin or DMSO for 5 days. The concentration of DMSO was kept low and constant between samples (0.1%, *v*/*v*). Thiazolyl Blue Tetrazolium Bromide (MTT, Sigma Aldrich) was added for 60 min to a final concentration of 0.5 mg/mL. The medium was carefully removed and MTT crystals were dissolved in isopropanol supplemented with 40 mM HCl. The absorbance was measured with Infinite 200 microplate reader (Tecan Group Ltd., Männedorf, Swizterland) at 570 nm with the reference wavelength 650 nm for background subtraction.

For data analysis, a dose-response fitting software, Dr-Fit, was used [26]. The analysis was performed with the “Automatic” fitting type, allowing the software to rank all four different fitting models (Hill equation, biphasic—two points of inflection, biphasic—with stimulatory effect, and triphasic—two points of inflection and stimulatory effect). “Standard deviation” weighting method and “Trust-region-reflective” fitting algorithm was used, as suggested by the software designers. The preferred fitting model was chosen based on the Bayesian Information Criterion (BIC), since it penalizes over-fitting stronger than other ranking criteria [26].

### 4.6. Treatment-Recovery Assay

The cells were seeded on transparent 96-well plates (Eppendorf, Hamburg, Germany). The next day the cells were either washed and fixed with 70% ethanol (to quantify the initial cell numbers) or treated with DMSO or 5 µM idasanutlin for one of the following time periods: (a) three days in the presence of DMSO (three days control); (b) three days in the presence of idasanutlin (three days treatment); (c) as in b plus three days in the presence of a fresh idasanutlin portion (six days treatment); (d) as in b plus wash with growth medium and an additional seven days of culture in fresh growth medium (three days treatment + seven days recovery); (e) as in c plus wash with growth medium and an additional seven days of culture in fresh growth medium (six days treatment + seven days recovery).

Following the treatments, the cells were washed, fixed with 70% ethanol, and stained with 2 µg/mL of Hoechst 33342 (Life Technologies, Carlsbad, CA, USA). The cells were washed and imaged using IX51 fluorescence microscope (Olympus, Tokyo, Japan). The experiment was performed three times (*n* = 3) with three images captured for every well in every experiment. The cell numbers were calculated with the use of ImageJ software [56] by the analysis of nuclei numbers in every image. The mean cell number per one picture for the time point 0 was 700–1500, depending on the cell type.

### 4.7. Colony Formation Assay

For the colony formation assay, the cells were treated with DMSO, 5 µM idasanutlin or 1 µM etoposide for four days. The cells were then harvested by trypsinization, seeded at low confluency (500 cells per well) on six-well plates and cultured for an additional seven days in a fresh medium devoid of treatment agents. The cells were washed twice with PBS, fixed with 70% ethanol, and stained with 0.5% crystal violet (*w*/*v*). Following thorough washing with deionized water, the plates were imaged with the ChemiDoc MP system (Bio-Rad Laboratories, Hercules, CA, USA) and analyzed using the ImageJ software [56] using the “Analyze particles” tool. The surviving fraction (SF) was calculated using the equation: SF = (PE _of treated sample_/PE _of control_) × 100%, where PE (plating efficiency) = N _colonies_/N _cells plated_.

### 4.8. Staining of Proliferating Cells

For the visualization of proliferating cells (idasanutlin-resistant clones) among cell cycle-arrested cell populations, BrdU labeling on culture plates was performed. U-2 OS cells were seeded on 6-well plates (5 × 10^5^ cells per well) and treated with 5 µM idasanutlin for 12 days. The medium was exchanged to fresh medium containing idasanutlin every 2–3 days. BrdU (10 µM) was added to the medium for the last 24 h of the treatment to allow for its incorporation into the DNA of every proliferating cell. Then, the cells were washed with PBS and fixed with 70% ethanol for 20 min at room temperature. Ethanol was removed and the cells were incubated with 2M HCl/0.5% Triton X-100 for 30 min, followed by a wash with 0.1 M Na_2_B_4_O_7_, pH 8.5. For BrdU staining, PBS containing 0.5% Tween 20, 1% BSA and 5 µL/mL of FITC-conjugated anti-BrdU antibody (BioLegend, cat. 364104) was added to each well and the plates were incubated at 4 °C overnight with gentle shaking (200 rpm). The BrdU-staining solution was then removed and the cells were incubated with a water solution of Hoechst 33342 (2 µg/mL) for 10 min. The wells were washed with distilled water and Fluoroshield Mounting Medium (Sigma Aldrich) was applied to protect fluorescent dyes from bleaching. Just before the imaging, the wells were again washed with distilled water and imaged with a Leica DMI6000B (AF7000) fluorescence microscope system (Leica, Microsystems, Wetzlar, Germany) using Tile Scan motorized stage application of Leica Application Suite X (LAS X) software to allow for the acquisition of the overview images of the whole surface of each well. For each well, the number of BrdU-labelled colonies and the total number of cells (number of Hoechst-stained nuclei, (5.5 ± 0.33) × 10^5^ on average) were calculated.

### 4.9. DNA Profiling (Cell Line Authentication)

Cell line authentication was performed by multiplex analysis of short tandem repeats (STR) at 15 loci (D7S820, CSF1PO, D13S317, TPOX, D5S818, D3S1358, D19S433, D2S1338, D16S539, D18S51, TH01, vWA, D21S11, D8S1179, and FGA) and Amelogenin, with the use of an Identifier Plus PCR amplification kit (Applied Biosystems, Foster City, CA, USA) and an ABI 3130xl Genetic Analyser (Applied Biosystems).

### 4.10. DNA Sequencing

Cell pellets were suspended in 400 μL of saline and DNA was isolated using the NucleoSpin Blood kit (Macherey-Nagel, Düren, Germany), according to the Manufacturer’s protocol. The obtained DNA was quantified spectrophotometrically (BioPhotometer, Eppendorf with Hellma adapter, Hellma Analytics, Müllheim, Germany).

Exons 5–8 of the *TP53* gene (LRG_321 t1) were sequenced, using exon-specific primers: exons 5–6—CTTTATCTGTTCACTTGTGCCC and TCAAATAAGCAGCAGGAGAAAG, exon 7—CTGCTTGCCACAGGTCTCC and TGATGAGAGGTGGATGGGTAG, exon 8—GGGACAGGTAGGACCTGATTTC and GGCATTTTGAGTGTTAGACTGG. First, the exon of interest was amplified using HotStarTaq DNA Polymerase (Qiagen, Hilden, Germany), according to the manufacturer’s recommendations, with an annealing temperature of 59 °C. After agarose gel electrophoresis confirming successful amplification, products were purified using the NucleoSpin^®^ Gel and PCR Clean-up kit (Macherey-Nagel). The products were quantified spectrophotometrically.

The sequencing reaction mix was prepared as follows. The final volume of 10 μL contained 2 μL polymerase mix (BigDye^®^ Terminator v3.1 Cycle Sequencing Kit, Thermo Fisher Scientific, Waltham, MA, USA), 2 μL reaction buffer (BigDye^®^ Terminator 5× Sequencing Buffer, Thermo Fisher Scientific), 1.6 μL of 1 μM forward or reverse primer, and 25 ng of the purified PCR product. All analyzed samples were sequenced in both directions—forward and reverse. Steps of the sequencing PCR: 96 °C for 1 min, 25 cycles comprised of 96 °C for 15 s, 55 °C for 7 s, 60 °C for 5 min. The obtained products were purified by ethanol precipitation: 2 μL sodium acetate/EDTA (1.5 M sodium acetate, pH  >  8.0 and 250 mM EDTA) was added to each sample, followed by DNA precipitation with 80 μL of 95% ethanol (EtOH). The samples were mixed thoroughly and centrifuged for 15 min at 10,000 × *g*. After removal of the supernatant, the DNA pellet was rinsed with 70% EtOH and centrifuged briefly. The pellet was then air-dried and resuspended in 20 μL highly deionized formamide (Hi-Di™, Thermo Fisher Scientific) and immediately run on a sequencer (Genetic Analyzer 3500 Series (Hitachi) Applied Biosystems).

Sequences were aligned in the SeqScape v2.7 software (Applied Biosystems) against the TP53 reference sequence NC_000017 from the NCBI GenBank database. Fragments with identified indels were additionally visualized in FinchTV 1.4.0 (Geospiza, Inc., Seattle, WA, USA). The alignments were exported and BLAT-searched in the UCSC GenomeBrowser [57] on Homo sapiens genome assembly GRCh38 (hg38), released in December 2013 [58]. For information about the potential impact of the identified variants on protein function and clinical outcome, the NCBI ClinVar database [28] and the UMD TP53 Mutation Database [29] were searched. The impact of the identified indel mutation on protein function was assigned by *SIFT INDEL* prediction tool [30].

## 5. Conclusions

The optimization of chemical structures of MDM2 antagonists has led to the development of potent drug candidates, among which idasanutlin (RG7388) is one of the most active, and the only one that has entered phase III clinical trials, so far. However, although modern Mdm2 antagonists have much improved activities compared to their precursors (i.e., nutlin-3), they suffer from similar weaknesses, which are limited elimination of p53^wt^ cancer cells and the generation of p53-mutated drug resistant subpopulations. For this reason, the development of combined treatment strategies will most likely be crucial for the successful use of MDM2 antagonists in the clinics.

## Supplementary Materials

The following are available online at http://www.mdpi.com/2072-6694/10/11/396/s1, Figure S1. The expression of MDM2 and p21 proteins following the treatment of SJSA-1 and U-2 OS cells with idasanutlin; Figure S2. The metabolic activity and nuclei staining of idasanutlin-treated cells; Table S1. The results of fitting of monophasic or multiphasic dose-response models using Dr-Fit software.
