# Supplementary material for: Prolonged Idasanutlin (RG7388) Treatment Leads to the Generation of p53-Mutated Cells
Source: Cancers (Basel). 2018 Oct 24;10(11):396. doi: 10.3390/cancers10110396 (PMC6266412; doi:10.3390/cancers10110396)
Supplement: Supplementary file 1 [file cancers-10-00396-s001.pdf]

Supplementary Materials: Prolonged Idasanutlin (RG7388) Treatment Leads to the Generation of p53-Mutated Cells

Lukasz Skalniak, Justyna Kocik, Justyna Polak, Anna Skalniak, Monika Rak, Agnieszka Wolnicka-Glubisz and Tad A. Holak

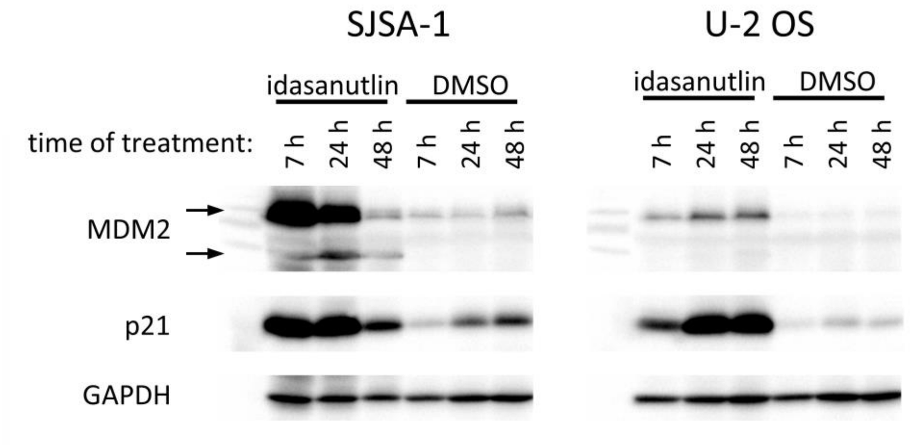

Figure S1. The expression of MDM2 and p21 proteins following the treatment of SJSA-1 and U-2 OS cells with idasanutlin. The cells were treated with 5 µM idasanutlin or equivalent volume of DMSO for 7, 24 or 48 hours, and western blot detection of MDM2 and p21 proteins was performed.

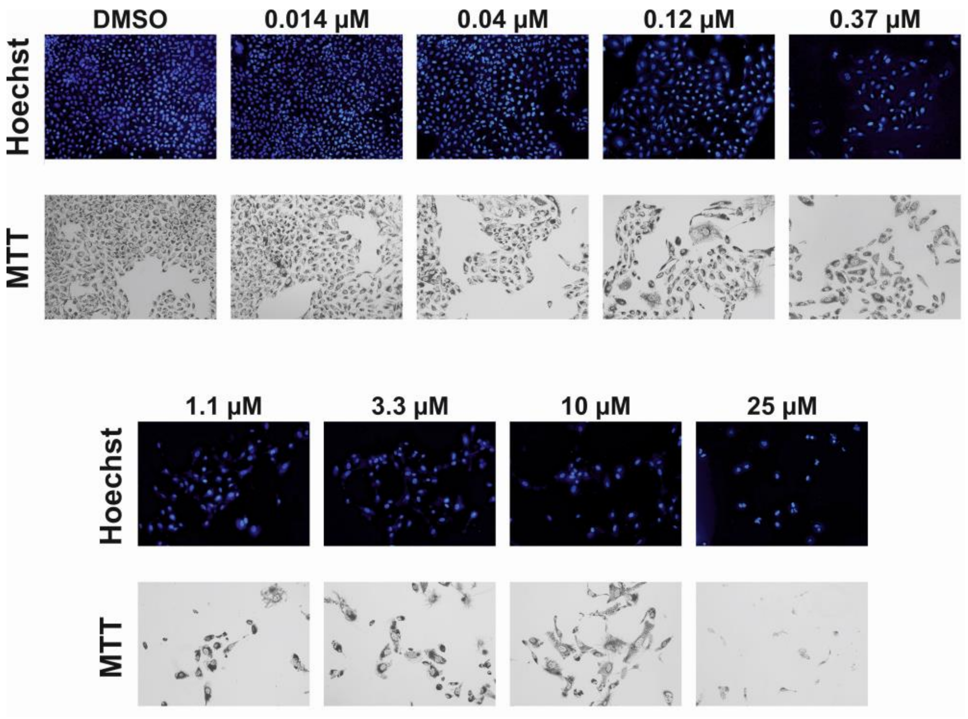

Figure S2. The metabolic activity and nuclei staining of idasanutlin-treated cells. U-2 OS cells were seeded at low confluency and treated for five days with increasing concentrations of idasanutlin or equivalent volume of DMSO. Then, the cells were stained with MTT to visualize metabolic activity, or fixed and stained with Hoechst 33342 to visualize cell nuclei. Up to the concentration of 10 µM idasanutlin-treated cells retain metabolic activity and integrity of the nucleus.

Table S1. The results of fitting of monophasic or multiphasic dose-response models using Dr-Fit software [1]. The fitting was performed on MTT results from RG7388-treated cells (see Figure 2a). For each cell line, three independent results were used. The analysis was performed in the "Automatic" mode, with "Standard deviation" weighting method and "Trust-region-reflective" fitting algorithm, as suggested by the software designers. The preferred model (highlighted in light grey) was chosen based on BIC value. GOF, goodness of fit, AIC, Akaike Information Criterion, BIC, Bayesian Information Criterion, and EC_{50}, the relative 50% effective concentration.

Cell Line	Model	GOF	AIC	BIC	EC_{50} [μM]		
					A	B	C
SJSA-1	Hill (1 inhib.)	0.260	51.014	111.014	-	0.019	-
	Biphasic (2 inhib.)	0.330	47.805	167.805	0.020	19.763	-
	Biphasic (1 inhib. + 1 stim.)	0.224	51.836	171.836	0.004	0.011	-
	Triphasic (1 inhib. + 2 stim.)	0.224	50.969	230.969	0.020	19.417	0.651
U-2 OS	Hill (1 inhib.)	<0.001	263.530	347.530	-	0.067	-
	Biphasic (2 inhib.)	0.567	56.410	224.410	0.043	16.569	-
	Biphasic (1 inhib. + 1 stim.)	<0.001	211.997	379.997	<0.001	0.013	-
	Triphasic (1 inhib. + 2 stim.)	0.109	76.417	328.417	0.043	10.766	43.465
MCF-7	Hill (1 inhib.)	<0.001	109.413	169.413	-	0.093	-
	Biphasic (2 inhib.)	0.591	40.210	160.210	0.092	34.184	-
	Biphasic (1 inhib. + 1 stim.)	0.002	85.062	205.062	0.002	0.024	-
	Triphasic (1 inhib. + 2 stim.)	0.439	44.235	224.235	0.043	37.631	0.015
SAOS-2	Hill (1 inhib.)	<0.001	225.517	309.517	-	15.804	-
	Biphasic (2 inhib.)	<0.001	226.400	394.400	9.986	25.174	-
	Biphasic (1 inhib. + 1 stim.)	0.001	113.623	281.623	0.466	14.025	-
	Triphasic (1 inhib. + 2 stim.)	<0.001	117.787	369.787	9.816	24.860	0.433

1. Di Veroli, G. Y.; Fornari, C.; Goldlust, I.; Mills, G.; Koh, S. B.; Bramhall, J. L.; Richards, F. M.; Jodrell, D. I. An automated fitting procedure and software for dose-response curves with multiphasic features. *Sci. Rep.* **2015**, *5*, 14701, doi:10.1038/srep14701.

© 2018 by the authors. Licensee MDPI, Basel, Switzerland. This article is an open access article distributed under the terms and conditions of the Creative Commons Attribution (CC BY) license (http://creativecommons.org/licenses/by/4.0/).
